# Lifestyle Behaviors and Physician Advice for Change Among Overweight and Obese Adults With Prediabetes and Diabetes in the United States, 2006

**Published:** 2011-10-15

**Authors:** Rashida Dorsey, Thomas Songer

**Affiliations:** Office of the Assistant Secretary for Planning and Evaluation; University of Pittsburgh, Pittsburgh, Pennsylvania.

## Abstract

**Introduction:**

The objective of this study was to examine the lifestyle behaviors of overweight and obese people with prediabetes or diabetes and to determine whether an association exists between reported behaviors and physician advice for behavior change.

**Methods:**

This investigation included overweight and obese people (body mass index ≥25.0 kg/m^2^) with prediabetes and diabetes aged 40 years or older identified from the 2006 National Health Interview Survey. Respondents reported attempts to control or lose weight, reduce the amount of fat or calories in their diet, and increase physical activity. Respondents also reported receipt of a physician recommendation for behavioral change in 1 or more of these areas. Data analysis included use of logistic regression stratified by sex and prediabetes/diabetes status to model odds of behavior by physician advice.

**Results:**

Most people reported trying to control or lose weight (prediabetes, 82%; diabetes, 75%). Fewer identified efforts to reduce the amount of fat or calories in their diet (prediabetes, 62%; diabetes, 71%) or increase physical activity (prediabetes, 53%; diabetes, 57%). Approximately one-third reported not receiving physician advice for each of these behavior changes. In logistic regression, physician advice for reducing the amount of fat or calories in the diet and increasing physical activity was generally associated with the reported corresponding behavior.

**Conclusion:**

Many respondents reported trying to control or lose weight, but fewer reported actually reducing fat or calories in their diet or increasing physical activity. Physician advice may influence attempts at behavior change among overweight and obese patients with prediabetes and diabetes.

## Introduction

The immense burden of diabetes underscores the utility and necessity of prevention. The findings of 2 landmark randomized clinical trials, the Diabetes Prevention Program (DPP), and the Look AHEAD study, provided evidence for the benefit of intensive modifications in lifestyle behaviors among people at high risk for diabetes as well as people with diabetes (1-3). In the DPP, weight loss was shown to be highly effective in reducing the incidence of diabetes among people at high risk for developing the disease. In the Look AHEAD study, weight loss was associated with improvements in diabetes control and reductions in cardiovascular disease risk factors. Thus, diabetes prevention efforts have a 2-pronged approach with a common strategy: primary prevention among people at risk for diabetes and secondary prevention among people with diabetes to prevent or delay the onset of complications, via weight loss (4).

Although clinical trials have demonstrated the benefits of weight loss among overweight and obese people either at high risk for diabetes or with diagnosed diabetes, few reports have described weight-loss behavior among these groups in the general population. Additionally, population-based reports have shown that the desire to achieve weight loss and the desire to create healthful changes overall occur at varying levels in the United States (5,6). Similarly, the literature has shown that among obese people, weight management behavior varies and that physician recommendation for weight loss is minimal, but few studies have focused on people with or at risk for developing diabetes (7,8). The objectives of this study were to 1) identify the prevalence of attempts to control or lose weight, reduce the amount of fat or calories in the diet, or increase physical activity reported and 2) examine physician recommendations to control or lose weight, reduce the amount of fat or calories in the diet, or increase physical activity, reported by overweight and obese people with prediabetes or diabetes.

## Methods

### Study design

This report is based on cross-sectional data available from the 2006 National Health Interview Survey (NHIS) (9). NHIS is an in-person survey of the US noninstitutionalized population in which participants are queried about their health and health care access and use. Participants are sampled using a complex multistage probability design, such that the sample is nationally representative. In 2006, a randomly selected adult aged 18 years or older per household also completed a more detailed interview that collected additional data related to health status and health behavior, including diabetes status, height, and weight. All NHIS data are self-reported. The total sample of the 2006 NHIS was 75,716 people, including 24,275 adults.

### Participants

This analysis included overweight and obese people with diabetes or prediabetes aged 40 years or older with at least 1 reported physician visit during the previous year. This investigation used height and weight to calculate body mass index (BMI). Recommendations from the US Dietary Guidelines define overweight as a body mass index (BMI) of 25.0 kg/m^2 ^to 29.9 kg/m^2^ and obese as 30.0 kg/m^2^ or more (10). The definition of diabetes was a self-report of physician diagnosis of diabetes. The definition of prediabetes was a self-report of physician diagnosis of impaired glucose tolerance, impaired fasting glucose, prediabetes, or borderline diabetes.

We focused on type 2 diabetes prevention initiatives; thus this study includes only people aged 40 years or older, the age group at highest risk for developing type 2 diabetes rather than type 1 diabetes. This analysis excluded people reporting no physician visits during the previous year (n = 74 among people with diabetes; n = 29 among people with prediabetes) to allow for an assessment of the association between physician advice and behavior change. This analysis also excluded pregnant women (n = 102 women with diabetes; n = 160 women with prediabetes) because of the different recommendations for lifestyle behavior change in this group. The final analytic sample consisted of 1,442 overweight and obese people with diabetes and 563 overweight and obese people with prediabetes.

### Measures

Respondents reported on their lifestyle behaviors, including whether they had done any of the following during the previous 12 months (yes/no): tried to control or lose weight, reduced the amount of fat or calories in their diet, or increased their physical activity. Respondents also reported whether they had received advice from a physician or health care provider to adopt any of these lifestyle behaviors during the previous 12 months (yes/no) (Appendix).

This investigation also examined additional variables to identify their role in the reported lifestyle behaviors and physician-recommended changes, including health insurance, health status, education, race/ethnicity, and diabetes treatment. The categories of health insurance were any coverage during the previous year or no coverage for the entire previous year. The categories of education were less than high school education; high school education or General Educational Development test, which is a high school diploma equivalency; or some college education or greater. The categories of race and ethnicity were non-Hispanic white, non-Hispanic black, Hispanic, and other racial/ethnic group. Among people with diagnosed diabetes, respondents reported current insulin use at the time of the survey. The categories of insulin use were users and nonusers. Categories of self-assessed health status were fair or poor, good, very good, or excellent. Additional covariates investigated included sex and age.

### Statistical analysis

We used SAS software version 9.1 (SAS Institute, Inc, Cary, North Carolina) to perform data management and SUDAAN version 9.0 (Research Triangle Institute, Research Triangle Park, North Carolina) to account for complex survey design and perform statistical analysis. This analysis used sample weights to account for differential probabilities of sample selection, nonresponse, and sample noncoverage. The level of significance for all analysis was *P* < .05. We stratified all analyses by prediabetes/diabetes status and sex. Because of the small sample size of stratified groups, this analysis grouped overweight and obese BMI categories together.

We calculated age-standardized prevalence estimates of respondent lifestyle behaviors and physician recommendation for lifestyle behaviors for the sample, which were standardized to the 2000 US population using the following age-groups: 40 to 49 years, 50 to 64 years, and 65 years or older. This investigation also used χ^2^ tests to assess differences in sex and demographic characteristics, lifestyle behaviors, and physician recommendation for lifestyle behaviors.

These analyses used logistic regression to model the odds of healthy lifestyle behavior by physician recommendation for lifestyle behavior change. We adjusted logistic regression models for age, race/ethnicity, education level, health insurance, and health status and stratified models by sex and prediabetes/diabetes status. Among people with diabetes, logistic regression models also included insulin use. For each model, the primary explanatory variable for all analyses was physician recommendation for lifestyle behavior change that corresponded to the specific respondent lifestyle behavior (eg, respondent report for increase in physical activity and physician advice for increase in physical activity). Among the sample with prediabetes, this investigation presents only logistic regression analysis modeling odds of increasing physical activity and reducing the amount of fat or calories in the diet. Because of small sample sizes, estimates from regression analysis modeling odds of trying to control or lose weight were statistically unreliable. We explored interactions between covariates and the advice explanatory variables but found no significant interactions.

## Results

The mean age for both prediabetic and diabetic men and women was 57 years (Table 1). The mean BMI was more than 30.0 kg/m^2^, the obese range, for all groups. Most respondents had a high school education or more. Reported health status was low; the majority of the men and women with prediabetes or diabetes identified their health as good, fair, or poor. Among people with diabetes, 20% of men and 30% of women reported the use of insulin (data not shown).

Overall, among people with prediabetes in this sample, 82% reported trying to control or lose weight, 62% reduced the amount of fat or calories in their diet, and 53% increased physical activity (data not shown). Among people with diabetes in the sample, 75% reported trying to control or lose weight, 71% reduced the amount of fat or calories in their diet, and 57% increased physical activity (data not shown). For both prediabetes and diabetes, a higher proportion of women than men reported trying to control or lose weight (range, 70%-86%) (Table 2). Diabetic women had a higher reported prevalence of reducing the amount of fat or calories in their diet than diabetic men (range, 55%-75%). Increasing physical activity was reported by fewer respondents (range, 53%-59%), and no sex difference was observed in either group. Approximately two-thirds of the respondents indicated that a physician had advised them to control or lose weight, reduce the amount of fat or calories in their diet, or increase physical activity (Table 2).

Among women with prediabetes, women reporting physician advice to increase physical activity were more likely to report an increase in physical activity compared to women with prediabetes who did not report receiving physician advice (Table 3). Men with prediabetes were more likely to report an increase in physical activity with physician advice, but no significant association was found between self-report of reducing the amount of fat or calories in their diet and physician advice for this behavior in this group. In addition, the association between physician advice for lifestyle behavior and the reported lifestyle behaviors of the respondents was significant for both men and women with diabetes for all 3 behaviors. The magnitude of association between advice and report of behavior change among men with diabetes was stronger than among women with diabetes.

## Discussion

In this cross-sectional analysis, approximately 82% of prediabetic and 75% of diabetic overweight and obese people reported trying to control or lose weight, but fewer reported reducing the amount of fat or calories in their diet (approximately 62% for prediabetic and 70% for diabetic respondents) or increasing physical activity (approximately 53% for prediabetic and 56% for diabetic respondents). In this study, which presents some of the first population-level estimates of lifestyle behavior and recommendations for behavior for these groups, 58% to 68% of the sample reported receiving a physician recommendation for weight loss, increasing physical activity, or reducing the amount of fat or calories in their diet. This research highlights that despite the advantages of weight loss that can be achieved through lifestyle modification, many overweight and obese people with prediabetes and diabetes do not report behaviors conducive to weight loss or physician advice for such behaviors.

The findings of this investigation are consistent with prior research. One study using data from the National Health and Nutrition Examination Survey reported weight-loss attempts for more than half of overweight and obese adults, and the Behavioral Risk Factor Surveillance System (BRFSS) reported estimates of weight-loss attempts of 60% to 72% for people with diabetes (11,12). The reported prevalence of physician advice also corresponds to prevalence estimates found in the general US population (8,13-15). Another analysis using data from BRFSS found that 42% of obese respondents in the general population reported physician advice for weight loss, but that prevalence rose to 61% among people with diabetes (13). This report, coupled with previous studies, reinforces the need for continued efforts to promote the adoption of and physician advice for lifestyle behavior change for overweight and obese people with prediabetes and diabetes.

Self-reported receipt of physician advice for lifestyle behaviors was strongly correlated with reported attempts to change lifestyle behaviors in this sample. Cross-sectional correlations were noted between reported behaviors (attempting to control or lose weight, reduce fat or calories in the diet, increase activity) and physician recommendations to do so; however it was not possible to identify whether physician advice precipitated behavior. Previous reports from the general population have shown a positive association between receipt of advice for lifestyle behaviors among the obese and adoption of the behavior; these are consistent with the findings of our study, which focused on people with prediabetes and diabetes (8,16,17). Likewise, although physician advice is significant, successful weight loss and weight control and sustained lifestyle behaviors are predicated upon many factors, including behavioral interventions, social support, and patient education. Advice from physicians for lifestyle behavior is a key first step of provider involvement to promote weight loss, but physician involvement is also important in tailored weight-management interventions, and physicians can help patients reduce barriers to achieving weight loss (18-23).

Physician counseling can be a vital part of weight management therapy (24,25). Each encounter physicians have with obese and overweight patients is a potential occasion to counsel weight loss (24). Approximately one-third of the overweight and obese respondents in our study did not report receiving recommendations to adopt these behaviors from their physicians. This suggests that physicians may be missing opportunities to offer lifestyle change advice. A few studies have identified barriers to physicians' providing advice for weight loss and lifestyle behavior change among their overweight and obese patients, which include lack of time during the office visit, training, and attitude toward obesity (23,26). Further studies may need to investigate strategies to reduce barriers to physicians providing lifestyle advice to their patients.

This study has a few limitations. Study variables were all self-reported; thus, recall bias is possible. Physician recommendation for lifestyle behaviors were reported by study respondents and not their physician; therefore, respondents may have reported not receiving advice when they actually did receive it. Data from a nationally representative provider-based survey have reported estimates of physician advice for physical activity and diet to obese patients somewhat lower than the current study; thus it is also possible for our respondents to have overreported receipt of physician advice (27,28). Additionally, because this is a cross-sectional study, it is not possible to assign direct causation between reported advice and reported behavior changes. Other factors may influence our findings that we were unable to measure. For instance, it was not possible to assess the nature and frequency of advice for lifestyle behavior change respondents received from physicians or the respondents' exposure to other lifestyle change strategies. BMI based on self-reported height and weight was used to classify the measured weight status of respondents. Self-reported height and weight data are often misreported (29). Finally, NHIS is limited to civilian, noninstitutionalized adults and does not include people living in nursing homes or other institutions. Thus, the findings of this study are generalizable to the overweight and obese noninstitutionalized civilian population with prediabetes and diabetes in the United States.

Appropriate lifestyle behaviors (which are important in both preventing diabetes and improving disease management and outcomes) and physician advice to adopt these behaviors are not reported by all overweight and obese adults with prediabetes and diabetes. Respondents who noted physician advice for these important behavioral changes also reported attempts to improve lifestyle behaviors more often than those not receiving this advice. Active physician involvement in obesity management for people with prediabetes and diabetes may be an overlooked strategy for population approaches to prevent diabetes and improve outcomes for people with diabetes.

## Figures and Tables

**Table 1:** Characteristics of Overweight and Obese Adults With Prediabetes and Diabetes, National Health Interview Survey, 2006^a^

Characteristic	**Prediabetes**			**Diabetes**		

	**Men, % (95% CI)^b^ (n = 228)**	**Women, % (95% CI)^b^ (n = 335)**	***P* Value^c^**	**Men, % (95% CI)^b^ (n = 667)**	**Women, % (95% CI)^b^ (n = 775)**	***P* Value^c^**
**Age, mean (SE), y^d^**	57.0 (0.3)	56.9 (0.3)	.07	57.5 (0.2)	57.9 (0.2)	.05
**BMI, mean (SE), kg/m^2^**	31.5 (0.6)	31.7 (0.4)	.23	32.3 (0.3)	35.0 (0.5)	<.001
**Race/ethnicity**						
Non-Hispanic white	60.1 (51.8-68.9)	49.8 (42.9-56.7)	.12	59.3 (54.0-64.3)	55.5 (50.2-64.3)	.80
Non-Hispanic black	13.3 (12.5-18.4)	17.0 (12.8-22.9)		13.5 (10.4-17.4)	17.3 (12.9-22.7)	
Hispanic	21.5 (15.7-30.0)	23.9 (17.9-31.0)		21.4 (17.4-26.0)	21.8 (17.1-27.3)	
Other racial/ethnic group	NR^d^	9.3 (6.1-13.4)		5.8 (3.9-8.5)	5.4 (3.7-8.0)	
**Education level**						
Less than high school	24.5 (17.3-32.5)	26.4 (20.8-32.9)	.37	25.4 (21.5-31.9)	23.6 (19.1-27.5)	.60
High school or equivalent	23.5 (16.8-30.7)	26.5 (20.8-33.1)		26.0 (21.3-30.7)	28.0 (21.2-30.9)	
Some college education or greater	52.1 (43.9-61.8)	47.1 (39.7-54.7)		46.6 (42.5-53.2)	48.4 (45.8-56.6)	
**Has health insurance**	83.4 (75.8-88.9)	83.0 (78.1-87.0)	.69	81.9 (77.3-85.7)	82.4 (77.9-86.2)	.74
**Health status**						
Fair or poor	24.1 (17.4-32.3)	28.2 (22.8-34.3)	.03	26.5 (21.9-31.6)	33.0 (27.6-38.9)	.30
Good	33.0 (23.9-43.4)	27.0 (21.3-33.5)		32.0 (27.5-36.9)	25.7 (21.7-30.1)	
Very good	33.4 (25.2-42.8)	28.0 (21.8-35.0)		31.0 (26.2-36.3)	29.8 (24.4-35.8)	
Excellent	NR^e^	16.8 (13.0-21.6)		10.5 (8.2-13.6)	11.5 (8.9-14.6)	

Abbreviations: CI, confidence interval; SE, standard error; BMI, body mass index; NR, not reported.

a Age-standardized to the 2000 US population.

b Values are % (95% CI) except where otherwise indicated.

c Calculated by using χ^2^ test.

d Not age-standardized.

e Unreliable cell estimates; relative SE >30%.

**Table 2:** Prevalence of Lifestyle Behavior Change Among Overweight and Obese Adults With Prediabetes and Diabetes, National Health Interview Survey, 2006^a^

Characteristic	**Prediabetes**			**Diabetes**		

	**Men, % (95% CI) (n = 228)**	**Women, % (95% CI) (n = 335)**	***P* Value^b^**	**Men, % (95% CI) (n = 667)**	**Women, % (95% CI), (n = 775)**	***P* Value^b^**
Tried to control or lose weight	78.5 (71.5-84.2)	85.5 (80.5-89.4)	.03	69.5 (64.5-74.0)	79.9 (76.2-83.2)	<.001
Increased physical activity	54.8 (46.0-63.3)	52.7 (45.3-59.9)	.68	54.8 (49.3-60.2)	58.5 (53.7-63.1)	.19
Reduced the amount of fat or calories in diet	55.1 (46.0-63.9)	66.1 (59.8-71.8)	.08	66.5 (61.8-70.9)	74.8 (70.6-78.6)	<.001
Physician advised to control or lose weight	68.7 (60.2-76.1)	65.5 (58.7-71.3)	.69	59.8 (55.1-64.2)	64.0 (58.8-68.8)	.13
Physician advised increase in physical activity	63.6 (55.1-71.3)	65.5 (58.2-71.9)	.45	62.9 (58.0-67.5)	65.5 (62.5-72.1)	.09
Physician advised reducing the amount of fat or calories in diet	61.7 (52.7-70.0)	62.8 (55.6-69.5)	.58	58.3 (53.4-63.1)	61.5 (56.2-66.6)	.10

Abbreviation: CI, confidence interval.

a Age-standardized to the 2000 US population.

b Calculated by using χ^2^ test.

**Table 3:** Odds of Lifestyle Behaviors Among Overweight and Obese Adults With Prediabetes and Diabetes, by Physician Recommendation for Behavior Change, National Health Interview Survey, 2006^a^

**Characteristic**	**Trying to Control or Lose Weight, OR (95% CI)^b^**	**Reducing Fat or Calories in Diet, OR (95% CI)^c^**	**Increasing Physical Activity, OR (95% CI)^d^**
Men with prediabetes	NC^e^	1.7 (0.8-3.9)	3.6 (1.5-8.7)
Women with prediabetes	NC^e^	2.8 (1.5-5.3)	3.0 (1.5-5.9)
Men with diabetes	9.1 (5.4-15.4)	11.3 (6.7-19.0)	4.6 (2.9-7.4)
Women with diabetes	4.1 (2.5-6.6)	5.7 (3.7-8.6)	2.8 (1.8-4.4)

Abbreviations: OR, odds ratio; CI, confidence interval; NC, not calculated.

a Models adjusted for age, race/ethnicity, education level, health insurance status, health status, and insulin use (diabetes only).

b Among people who reported receiving physician advice to control or lose weight compared to those who reported not receiving advice.

c Among people who reported receiving physician advice reduce fat or calories in diet compared to those who reported not receiving advice.

d Among people who reported receiving physician advice to increase physical activity compared to those who reported not receiving advice.

e Because of small sample sizes, values were statistically unreliable among the prediabetes sample and therefore are not presented.

## Appendix: Survey Questions From the 2006 National Health Interview Survey

### Sample Adult Component: Health Conditions

Doctors and other health professionals often advise patients on ways they can lower their risk for health problems and/or certain diseases. For the next 3 questions, please think about their advice IN THE PAST 12 MONTHS.

DURING THE PAST 12 MONTHS, have you been told by a doctor or health professional to ... control your weight or lose weight?

Yes

No

Refused

Don't know

DURING THE PAST 12 MONTHS, have you been told by a doctor or health professional to increase your physical activity or exercise?

Yes

No

Refused

Don't know

DURING THE PAST 12 MONTHS, have you been told by a doctor or health professional to … reduce the amount of fat or calories in your diet?

Yes

No

Refused

Don't know

People often engage in activities to lower their risk for health problems and/or certain diseases. For the next 3 questions, please think about what YOU have been doing IN THE PAST 12 MONTHS.

DURING THE PAST 12 MONTHS, have you … been trying to control your weight or to lose weight?

Yes

No

Refused

Don't know

DURING THE PAST 12 MONTHS, have you ... increased your physical activity or exercise?

Yes

No

Refused

Don't know

DURING THE PAST 12 MONTHS, have you … reduced the amount of fat or calories in your diet?

Yes

No

Refused

Don't know
